# Endocannabinoid administration affects taste preference and the expression of cannabinoid and opioid receptors in the amygdala of early lactating cows

**DOI:** 10.1038/s41598-023-31724-3

**Published:** 2023-03-27

**Authors:** Jessica Schwerdtfeger, Annika Krause, Claudia Kalbe, Gemma Mazzuoli-Weber, Anja Eggert, Birger Puppe, Björn Kuhla, Volker Röttgen

**Affiliations:** 1 Institute of Nutritional Physiology ‘Oskar Kellner’, Research Institute for Farm Animal Biology (FBN), Wilhelm-Stahl-Allee 2, 18196 Dummerstorf, Germany; 2 Institute of Behavioural Physiology, Research Institute for Farm Animal Biology (FBN), Wilhelm-Stahl-Allee 2, 18196 Dummerstorf, Germany; 3 Institute of Muscle Biology and Growth, Research Institute for Farm Animal Biology (FBN), Wilhelm-Stahl-Allee 2, 18196 Dummerstorf, Germany; 4 Institute for Physiology and Cell Biology, University of Veterinary Medicine, 30173 Hannover, Germany; 5 Institute of Genetics and Biometry, Research Institute for Farm Animal Biology (FBN), Wilhelm-Stahl-Allee 2, 18196 Dummerstorf, Germany; 6 Behavioural Sciences, Faculty of Agricultural and Environmental Sciences, University of Rostock, Justus-Von-Liebig-Weg 6B, 18059 Rostock, Germany

**Keywords:** Neuroscience, Physiology

## Abstract

The aim of the study was to investigate the influence of intraperitoneal *N*-arachidonoylethanolamide (AEA) on taste preference for feed and water, tongue taste receptor signalling (*TAS1R2*, *GNAT3*), and endocannabinoid (*CNR1*, *CNR2*, *GPR55*) and opioid (*OPRD1*, *OPRK1*, *OPRM1*, *OPRL1*) receptors in the amygdala and nucleus accumbens in periparturient cows. We conducted taste preference tests using unaltered, umami-tasting, and sweet-tasting water and feed, before and after calving. After calving, eight cows received AEA injections (3 µg/(kg bodyweight × day), 25 days), whereas eight control (CON) cows received saline injections. Tissue was sampled 30 days after calving. Before calving, both cow groups preferred sweet-tasting feed and umami-tasting water. After calving, only the AEA-treated group preferred sweet-tasting feed, whereas the CON group showed no clear taste preference. In the amygdala, the mRNA expression of *CNR1*, *OPRD1* (left hemisphere) and *OPRK1* (right hemisphere) was lower in AEA animals than in CON animals, whereas no differences were found in the nucleus accumbens and tongue taste receptor expression. In conclusion, AEA administration enhanced existing taste preferences and reduced the expression of specific endocannabinoid and opioid receptors in the amygdala. The results support endocannabinoid-opioid interactions in the control of taste-dependent feed preference in early lactating cows.

## Introduction

The sense of taste plays a pivotal role in identifying the nutrient content of feed. The five basic tastes (sweet, umami, salty, bitter and sour) are indicators of different feed compositions^1^. For example, sugar- and starch-rich diets are perceived as sweet, whereas protein-rich diets elicit more umami taste^1^. The taste enables animals to select feed according to their nutritional needs and avoid the ingestion of harmful or even toxic feedstuff^1^. Various chemoreceptors, so-called taste receptors, expressed in taste buds on the tongue, can detect the five basic tastes, and cows are able to discriminate between these tastes^1–3^.

Signals from taste receptors are conveyed to the brain and processed in gustatory cortex areas^4^. Furthermore, taste signals are also transduced to the limbic system^4^. The direct involvement of the limbic system shows that feed consumption is connected to the endogenous reward system, eliciting hedonic responses. The reward system, among others, motivates animals to ingest feed with a certain taste^5^. Feed rewards and feed intake itself are modulated by the endocannabinoid and opioid systems^6–9^. For example, administration of the endocannabinoid *N*-arachidonoylethanolamide (AEA) is known to increase feed intake^10^, hedonic eating, and the consumption of sweetened water in rodents^11,12^. It has also been shown that intraperitoneal (i.p.) administration of AEA reduces stress-induced hypophagia and increases the short-term feed intake of dairy cows^13,14^. AEA acts as a partial agonist of the cannabinoid receptor type 1 *(CNR1*)^15,16^, type 2 (*CNR2*)^15,17,18^ and G protein-coupled receptor 55 (*GPR55*)^19,20^. These receptors are also expressed in brain areas associated with feed reward processes alongside opioid receptors, e.g., in the amygdala and nucleus accumbens (NAc)^21–24^. There are clear hints that the opioid system is involved in feed intake regulation and feed preference in bovines. Montoro et al. reported a reduced preference for sweet-tasting feed and reduced feed intake after administration of the opioid receptor antagonist naloxone in calves^25^. In addition to AEA and naloxone, other factors, such as the hormonal (e.g. leptin) and gestational status and previous experiences of an animal, can alter feed preference and feed intake^26–28^. As an example, small ruminants may learn to avoid prior preferred feedstuff if they suffer later from physical discomfort as a postingestive consequence^29^.

To investigate the influence of substances such as AEA on taste preferences, taste preference tests are conducted. In those tests, animals are offered a choice between two or more different tasting matrices, e.g., feed and water, consecutively or simultaneously. However, the matrix offered to the animals must be carefully designed, as each feed component, such as silage, hay, grain, etc., has its own taste, whereas drinking water is usually tasteless. The general readouts of a taste preference test are feed intake, time spent feeding, or more detailed behavioural measures, e.g., order of choice or orofacial expressions^30,31^. In cattle, several taste preference tests have been performed, some of them using complex tasting agents (e.g., anise, vanilla)^32,33^, while others used substances having basic tastes (e.g., sugar, monosodium glutamate)^3,33^. For early lactating dairy cows and calves, a sweet taste preference in feed and water has been demonstrated in several studies^3,33–36^. One study reported a preference for feed with unaltered or umami taste over feed supplemented with anise, alfalfa or molasses in early lactating cows^33^. However, no knowledge exists about the taste preference of late gestating cows, and only one study tested the feed preference of periparturient cows for a flavoured diet by measuring dry matter intake (DMI)^37^.

We hypothesize that taste preference changes from late gestation to early lactation and that AEA administration modulates the taste preference of early lactating dairy cows. Thus, the aims of the present study were (i) to investigate the taste preference of late-pregnant dairy cows by offering sweet-tasting, umami-tasting and taste-unaltered feed and water and (ii) to examine whether AEA compared to NaCl (CON) administration in early lactation modifies taste preference, the expression of endocannabinoid receptors and taste-related genes in the tongue and the expression of opioid receptors in the amygdala and nucleus accumbens of dairy cows.

## Results

### Habituation and reminder

During habituation to the water preference test (WPT) setting, cows showed a higher relative total duration (TD) and relative total number (TN) at the lateral water bowl than at to the left and right water bowl (*P* < 0.001; Supplementary Figure 1a). However, the relative water intake from the left and right drinking bowl was not significantly different.

During the reminder, the cows showed no significant differences for usage of the left or right water bowl as assessed by relative TN and TD. However, cows consumed significantly more water from the right drinking bowl (*P* < 0.05, Supplementary Figure 1b).

During the habituation to the feed preferences test (FPT) setting, the cows showed no significant differences for relative DMI, TD and TN between the three feeding bins (Supplementary Figure 2a). During the reminder, cows placed their head significantly longer (TD) in and ingested significantly more dry matter from the left bin than from the middle bin (*P* < 0.05; Supplementary Figure 2b). In addition, cows consumed a greater portion of feed from the right bin than from the middle bin (*P* < 0.05). The ANOVA results are presented in Supplementary Table 1.

### Water preference test

**Figure 1:**
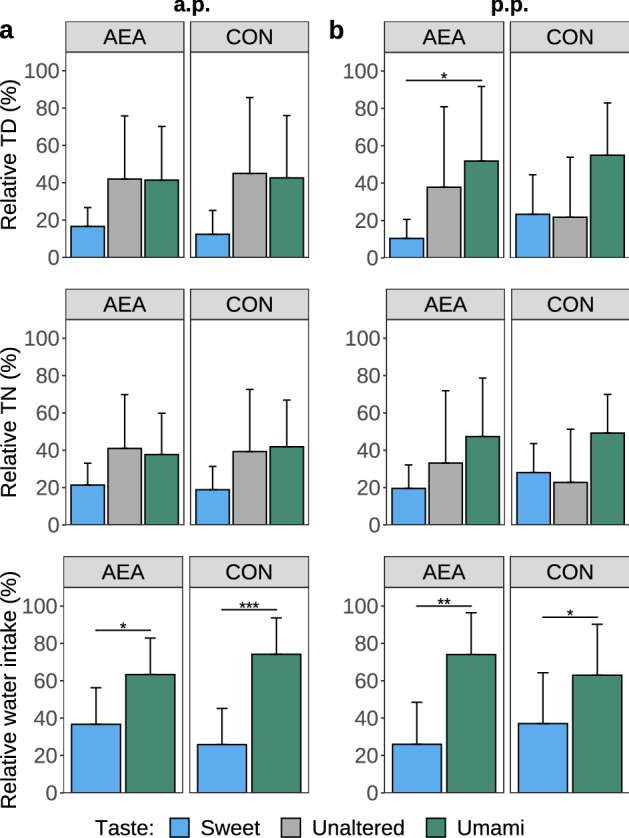
Three-day mean of percentage of total duration (TD) and total number (TN) of events the cow’s head entered the drinking bowl, and water intake of sweet and umami tasting or taste unaltered water during the water preference test (**a**) in the antepartum (a.p.) and (**b**) postpartum (p.p.) period. During the p.p. period, cows were treated intraperitoneally with NaCl (CON, n = 8) or *N-*arachidonoylethanolamide (AEA, n = 8). The mean of the observed data ± SD is shown for interpretation. *P < 0.05, **P < 0.01, ***P < 0.001.

In the antepartum (a.p.) period, the AEA group had comparable relative TD and TN at all three water bowls. Comparing sweet and umami tastes, AEA cows consumed 1.7 times more umami-tasting water than sweet-tasting water (*P* < 0.05; Fig. 1a). Similarly, CON cows showed no significant difference in the relative TN and TD at all three water bowls but consumed 2.9 times more umami-tasting water than sweet-tasting water (*P* < 0.001).

After parturition, the AEA group drank 2.8 times more umami-tasting water than sweet-tasting water (*P* < 0.01), while the CON group drank 1.7 times more umami-tasting water than sweet-tasting water (*P* < 0.05) (Fig. 1b). Accordingly, the AEA group showed a higher TD (*P* < 0.05) on umami-tasting water compared to sweet-tasting water (Fig. 1b). However, CON cows revealed no significant differences in relative TD and TN between the two tastes. The ANOVA results are presented in Supplementary Table 2.

### Feed preference test

**Figure 2:**
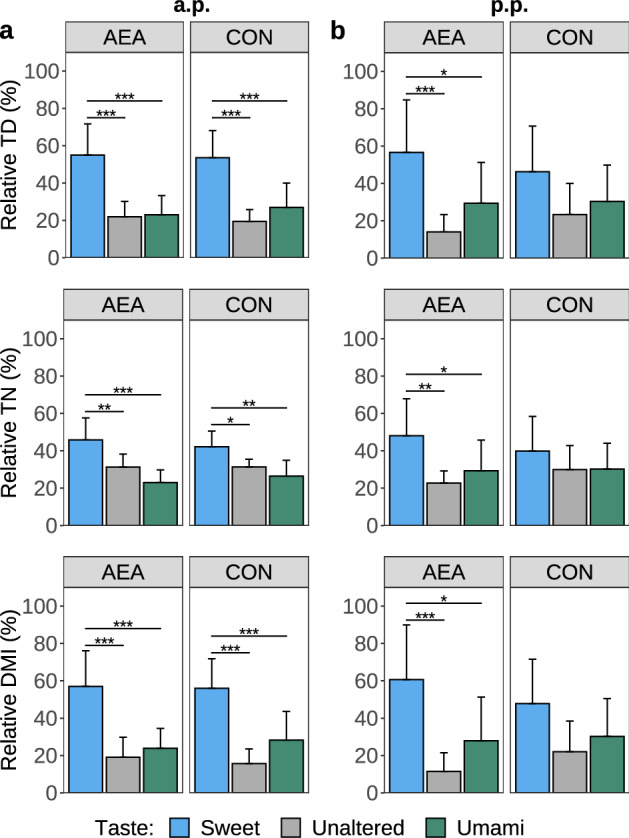
Three-day mean of percentage of total duration (TD) and total number (TN) of events the head entered the feeding bin, as well as dry matter intake (DMI) of the sweet and umami tasting or taste unaltered feed during the feed preference test (**a**) during the antepartum (a.p.) and (**b**) postpartum (p.p.) period. During the p.p. period, cows were treated intraperitoneally with NaCl (CON, n = 8) or *N*-arachidonoylethanolamide (AEA, n = 8). The mean of the observed data ± SD is shown for interpretation. *P < 0.05, **P < 0.01, ***P < 0.001.

The three-day average feed intake was not different between AEA and CON cows during the FPT, neither a.p. nor postpartum (p.p.) (Supplementary Figure 3). During the a.p. period, the TD, TN and DMI of sweet-tasting feed were significantly higher than those of control or umami-tasting feed in both the AEA and CON groups (*P* < 0.05; Fig. 2a). However, both groups revealed no significant differences in relative TD, TN and DMI between umami-tasting and control feed.

During the p.p. period, AEA cows revealed significantly higher relative TD, TN and DMI for sweet-tasting feed compared to control or umami-tasting feed (Fig. 2b). In contrast to the AEA group, the CON group showed no significant difference in relative DMI, TD, and TN for all three diets. The ANOVA results are presented in Supplementary Table 3.

### mRNA expression

**Figure 3:**
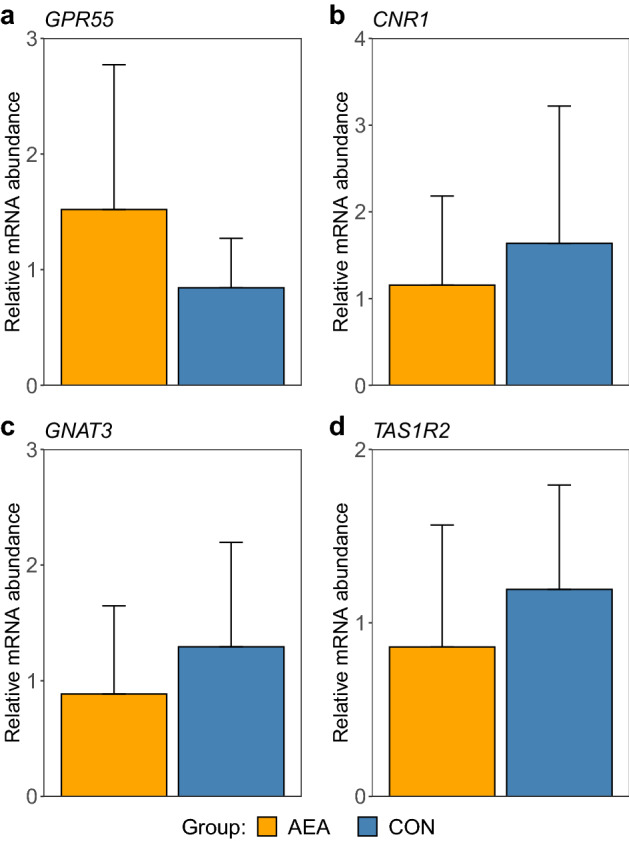
Relative mRNA abundance of (**a**) G protein-coupled receptor 55 (*GPR55*), (**b**) cannabinoid receptor 1 (*CNR1*), (**c**) G protein subunit alpha transducin 3 (*GNAT3*) and (**d**) taste receptor type 1 member 2 (*TAS1R2*) in tongue epithelia of cows treated intraperitoneally with NaCl (CON, n = 8) or *N*-arachidonoylethanolamide (AEA, n = 8). The mean of the observed data ± SD is shown for interpretation.

The *CNR1, GPR55, TAS1R2* and *GNAT3* mRNA expression levels in the tongue epithelium were not significantly different between the AEA and CON groups (Fig. 3). The ANOVA results are presented in Supplementary Table 4.

**Figure 4:**
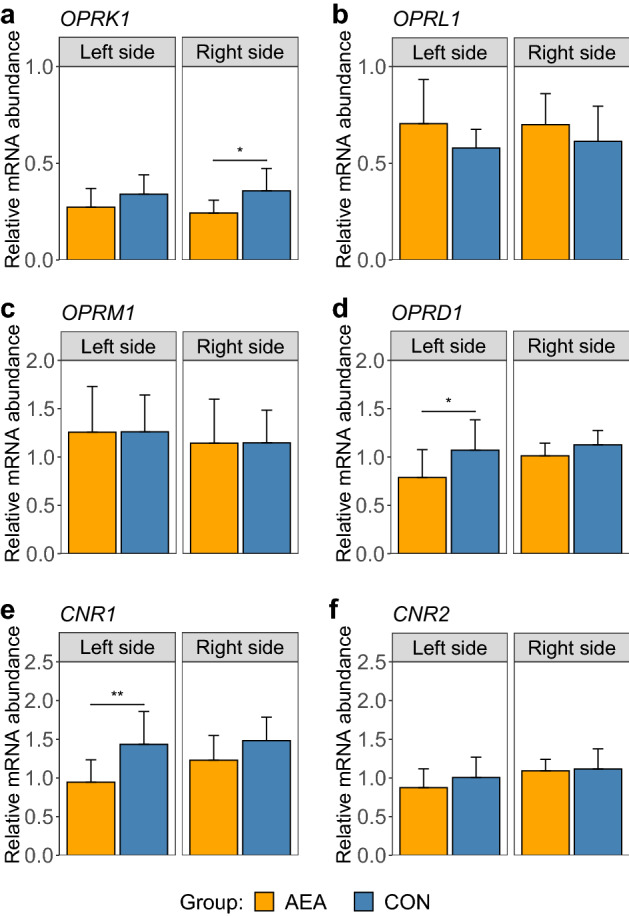
Relative mRNA abundance of (**a**) opioid receptor kappa 1 (*OPRK1*), (**b**) opioid related nociceptin receptor 1 (*OPRL1*), (**c**) opioid receptor mu 1 (*OPRM1*), (**d**) opioid receptor delta 1 (*OPRD1*), (**e**) cannabinoid receptor 1 (*CNR1*) and (**f**) cannabinoid receptor 2 (*CNR2*) in left and right amygdala of cows treated intraperitoneally with NaCl (CON, left hemisphere n = 8, right hemisphere n = 8) or *N*-arachidonoylethanolamide (AEA, left hemisphere n = 8, right hemisphere n = 8). The mean of the observed data ± SD is shown for interpretation. *P < 0.05, **P < 0.01.

The *CNR1* and opioid receptor delta 1 (*OPRD1*) mRNA expression levels in the left amygdala were lower in AEA cows than in CON cows (Fig. 4). In contrast, the mRNA expression of *CNR2*, opioid-related nociceptin receptor 1 (*OPRL1*), opioid receptor mu 1 (*OPRM1*), and opioid receptor kappa 1 (*OPRK1*) in the left amygdala did not differ between groups. In the right amygdala, however, the mRNA expression of *OPRK1* was lower in AEA cows than in CON cows, but the transcript levels of *CNR1, CNR2, OPRL1, OPRM1,* and *OPRD1* were unaltered between groups. Moreover, AEA treatment had no effect on the endocannabinoid and opioid receptor mRNA abundances in the right and left nucleus accumbens (Supplementary Figure 4). The ANOVA results are presented in Supplementary Tables 5 and 6.

### Plasma AEA and leptin concentration

Repeated AEA injections resulted in significantly higher plasma AEA concentrations at Day 21 p.p. (AEA: 0.174 ng/ml, CON: 0.098 ng/ml; *P* = 0.017, Supplementary Figure 5). However, the plasma leptin concentration was not significantly different between the two groups (*P* = 0.382; Supplementary Figure 5). The ANOVA results are presented in Supplementary Table 7).

## Discussion

In this study, we show that cows in late gestation preferred sweet-tasting feed and umami-tasting aqueous solutions. After calving, AEA-treated animals maintained their preference for sweet-tasting feed and umami-tasting water, whereas CON cows did not maintain their preference for sweet-tasting feed but did for umami-tasting water. These findings clearly demonstrate the taste preference change from late gestation to early lactation and the taste modifying influence of the endocannabinoid AEA.

During the a.p. FPT, cows of both groups significantly preferred sweet-tasting feed in all three measured parameters (DMI, TD, TN). This sweet taste preference was, to the best of our knowledge, only described for calves and early lactating dairy cows^25,33,34^. In the p.p. period, a clear preference for sweet-tasting feed could only be observed in the AEA group. Cows of the CON group did not show a clear taste preference p.p., as the TD, TN and DMI did not differ significantly between the three tastes offered. This finding is in contrast to previous studies demonstrating a preference for sweet taste (diets supplemented with sucrose) in early lactating cows^33,34^. The conflicting results may be due to the differences in time after calving, preference test design, duration and the concentration of tastants^33,34^. However, in the second experiment, Nombekela et al. found a clear preference for a diet supplemented with sodium glutamate (umami) compared to molasses (sweet) in early lactating cows^33^.

Our results further indicate that AEA promotes the preference for sweet-tasting feed. This finding is consistent with studies in rodents. Yoshida et al. reported an increased sweet taste preference after i.p. AEA application in mice^11^. In rats, AEA microinjection into the nucleus accumbens enhanced hedonic responses to a sweet taste^12^. Conclusively, it seems that AEA modifies the response to sweet taste, whereas the response to other tastes, e.g., umami, remains unchanged^11,12^.

During the a.p. WPT, neither cow group showed a clear taste preference. The relative TN and TD of the animals on umami-tasting water and taste-unaltered water were comparable, but they were numerically lowest for sweet-tasting water. The low preference for sweet-tasting water is surprising because cows preferred sweet feed in the a.p. FPT, and various studies have reported a preference in cattle for sweet solutions^3,36,38^. In our study, we wanted to achieve a high taste intensity, which was only possible with a nonnutritive sweetener due to the risk of ruminal acidosis. The low intake of sweetened water in our study could be due to the use of saccharin as a sweetener. Saccharin may evoke different signal transduction pathways. In mice, artificial sweeteners activate a different taste transduction pathway than natural sweeteners^39^ and recent studies in cattle^40–42^ support these findings. Furthermore, studies in humans suggested that saccharin, especially in high concentrations, evokes a bitter aftertaste, which is due to the additional activation of bitter taste receptors^43^. Bitter tasting solutions such as quinine solution have been shown to evoke even adverse effects in cattle^44^. This might explain the conflicting results of our FPT and WPT. Further explanations for the higher intake of umami water could be the choice of matrix. Water has only a weak intrinsic taste, whereas feed consists of several components with pronounced intrinsic taste.

During the p.p. WPT, both groups consumed more umami-tasting water than sweet-tasting water, and AEA-treated cows showed a higher TD for umami-tasting water. The latter finding can be interpreted as an indication of preference, as the animals were willing to spend more time consuming this taste.

The results obtained from the FPT and WPT support the hypothesis by Mahler et al., who suggested that AEA only reinforces the most preferred stimulus before treatment, while less preferred stimuli may remain unaltered^12^.

The position of the bin or bowl may also affect the choice of feed and water^45^. In our study, the bin and bowl positions did not influence choices, as TN, TD and DMI did not differ during the FPT and WPT habituation. The preference for the lateral drinker during WPT habituation cannot be interpreted as an expression of side preference because of the higher water flow rate. Cattle are suction drinkers and as such require a certain level of water around the muzzle^46^.

During the reminder, cows again showed no clear preference for a bowl position, except that they drank 1.6 times more from the right side. Furthermore, cows in early lactation preferred the left and right bins over to the middle bin for feed intake. The changes from habituation to the reminder suggest that cows have learned to associate the position of the bins or water bowls, respectively, with taste characteristics during the previous preference tests. Earlier studies demonstrated that cows are able to associate locations with the quality of feed^47,48^ and can remember the bin position^49,50^. Therefore, side preferences during the reminder of our study are presumably the result of the previous preference test.

In addition to the previously mentioned factors, further sensory properties of the feed influence feeding behaviour and taste preference. Cattle also use sight, smell, and haptics for feed selection^51,52^. In the current study, all measures were taken to influence taste solely and not the appearance, smell or haptics of the feed. Hence, we conclude that the choice of feed or water was primarily driven by taste.

The sense of taste allows the animals to assess the nutritive value of feed and therefore to select the feed according to their nutritional needs^53^. Sweet taste is one sign for edible feed and signals the presence of calories, even though sweetness does not necessarily reflect energy content^54,55^. It is conceivable that cows use sweet taste as an indication of the presence of rapidly fermentable carbohydrates. The observed preference for sweet-tasting feed a.p. could be due to the higher caloric content in comparison to the control feed or umami-tasting feed. In late gestation, high amounts of energy and protein are needed to meet the requirements for foetal development^56,57^. The sweet taste preference may promote the intake of energy dense feed in late gestation to ensure sufficient energy intake. Absence of preference for sweet-tasting feed in the CON group in the p.p. period is surprising, as the energy requirement increases rapidly due to the increase in milk production after calving. Thus, the intake of energy-dense feed should also be beneficial in this period. However, because the lactation diet contained 1.4 times more sugar than the close-up diet, it may be perceived as too sweet. A high density of rapidly fermentable sugars increases the production of short-chain fatty acids in the rumen and could lower the ruminal pH, which could result in ruminal acidosis. Therefore, a high sweet taste intensity may be a sign for ruminants to avoid negative physiological consequences. Zahorik et al. showed that ruminants avoid feed that is associated with illness and feed that causes physiological discomfort hours after ingestion^58^.

The change in taste preference from a.p. to p.p. could also be due to alterations in taste sensitivity. In humans and mice, it has been shown that taste changes during the course of pregnancy and in the postpartum period^59,60^. Some authors suggested that these alterations could be due to hormonal changes occurring relative to parturition^59–61^. The ability to modify taste has been demonstrated for leptin^62^, oxytocin^63^ and estrogen^64^ in rodents, but the influence of hormonal changes occurring in the peripartum period on taste sensitivity in cattle remains unclear. Leptin is further involved in the regulation of the AEA tone. In rodents, leptin reduces the AEA level in the hypothalamus^28^. In our study, the plasma leptin concentration did not differ between both groups on Day 21 p.p. Hence, the continuous preference for sweet tasting feed and in increased AEA plasma level in the treatment group was presumably driven by AEA treatment.

The specific mechanisms of peripheral taste sensation and taste transduction in cattle are not yet fully understood, but genes encoding sweet and bitter taste receptors and α-gustducin are evident in cow taste bud cells^65^. In the current study, the mRNA abundances of *TAS1R2*, *GNAT3,* and the endocannabinoid receptors *CNR1* and *GPR55* were not different between groups, suggesting that sweet taste signalling was not altered by AEA administration. Our findings are in contrast to results obtained in mice. In mice, the endocannabinoid system could alter peripheral sweet taste sensitivity, which is mediated by *CNR1* coexpressed with *TAS1R3*^11^. Administration of endocannabinoids increased the responses of the sweet taste receptor and chorda tympani nerve to a sweet-tasting solution, while the response of mice to sour, bitter, salty and umami tasting-solutions remained unaltered^11^. Furthermore, AEA-treated wild-type mice, in contrast to *CNR1*^-/-^ mice, showed an increased licking rate to a sweetened solution^11^. Although we observed a preference for sweet-tasting feed, the lack of changes in taste receptor expression after AEA administration might be because the tongue sampling was performed several days after the preference test when cows had no access to taste-altered feed.

The opioid and endocannabinoid systems of the NAc and amygdala are both involved in the feed rewarding process. In rodents, the application of a mu opioid receptor agonist into the NAc hot spot enhances “liking” and “wanting” reactions^66^. “Liking” refers to the hedonic impact and was measured by positive orofacial patterns, whereas “wanting” as incentive motivation was measured by feed intake^66^. In addition, application of a delta opioid receptor agonist into the NAc increased intake of a sweet-tasting solution in rats^67^, whereas administration of a kappa opioid receptor agonist did not alter or decreased feed or water intake^67,68^. In calves, blockade of opioid receptors with naloxone inhibited the sensorial pleasure elicited by sweet-tasting feed^25^. The endocannabinoid system may interact with the opioid system, as endocannabinoid receptors and mu opioid receptors are coexpressed in some neurons of the NAc shell and core^22^. Surprisingly, AEA administration had no effect on cannabinoid or opioid receptor mRNA expression in the NAc in our study, suggesting that AEA administration did not alter the reward process at the mRNA level. However, as described for the tongue, sampling of brain tissue several days after the FPT may explain the lack of expression differences. Another reason may be the functional heterogeneity of the NAc^69^, which could be paralleled by a different mRNA expression of opioid receptors in various subregions; however, our sampling method did not allow for a differentiation of subregions. On the other hand, unaltered mRNA expression of opioid receptors in the NAc is consistent with the finding that AEA does not alter the gene expression of proenkephalin, a precursor of various opioid peptides, in the NAc of rats^70^.

Administration of a mu receptor agonist into the amygdala enhances the “wanting” onto a specific reward cue in the central nucleus^71^, and kappa and delta opioid receptors and their ligands in the amygdala were described to be involved in rewarding processes^72,73^ and modulation of emotional processes^74–76^. Interestingly, the mRNA expression of *OPRD1* was downregulated in the left amygdala, whereas *OPRK1* expression was downregulated in the right amygdala. Downregulation of opioid receptor expression has also been observed after chronic exposure to an agonist^77,78^. It is known that the AEA analogue R-methanandamid increases the mRNA expression of proenkephalin^70^ and that proenkephalin-derived opioid peptides bind to delta, mu and kappa opioid receptors with different affinities^79^. Thus, it is conceivable that AEA may also increase the expression of endogenous proenkephalin, which in turn causes downregulation of *OPRD1* and *OPRK1* in the amygdala. However, the downregulation of the opioid receptors could also be caused by increased opioid release caused by positive emotions^80^. Koepp et al. showed that the activity of the amygdala is reduced during positive emotions mediated by increased opioid-related inhibition^80^. Kalbe and Puppe have demonstrated the downregulation of opioid receptors (*OPRK1* and *OPRD1*) at the mRNA level in the porcine amygdala as a consequence of an experimental food-rewarded learning paradigm^76^. This emphasizes the involvement of these opioid receptors in processes of emotionally driven motivational control and behavioural reinforcement, as we observed in a similarly interpretable manner for the enhanced sweet preference of AEA-treated animals in the present study. However, further investigations are needed to elucidate the lateralization of altered opioid receptor expression in the amygdala.

In the current study, AEA treatment resulted in a 1.78 times higher AEA plasma level. Hence, the reduced *CNR1* mRNA expression in the left amygdala could be due to chronic exposure to AEA, an agonist of *CNR1*. This assumption is supported by several studies showing a region- and time dependent alteration of *CNR1* gene expression and decreased receptor binding affinity following chronic exposure to *CNR1* agonists in rodents^81–83^. However, the amygdala is further involved in processing taste intensity and valence of feed^84^. *CNR1* downregulation in the left amygdala only indicates its involvement in the assessment of taste intensity. Veldhuizen et al. found that the left amygdala is involved in the central gain mechanism of taste in humans^85^. The central gain mechanism influences taste intensity perception by inhibitory outputs from the central medial nucleus of amygdala to the gustatory and limbic nuclei of the thalamus^85^. In our study, downregulation of *CNR1* in the left amygdala therefore indicates that chronic administration of AEA modulated the perception of taste intensity. The potential modified taste intensity perception could be an explanation for the persistent preference for sweet-tasting feed in AEA cows, despite the higher sugar concentration of the lactation diet. In contrast to *CNR1*, *CNR2* gene expression was not altered by chronic AEA administration in either the amygdala or NAc, suggesting that the modulation of taste perception intensity is primarily mediated by *CNR1*. In conclusion, the present study shows that cows in late pregnancy preferred sweet-tasting feed, whereas no clear taste preference could be detected during the WPT in the a.p. period. In early lactation, AEA promoted the preference for sweet-tasting feed. However, AEA administration did not alter the mRNA expression of taste receptors in the tongue or opioid and endocannabinoid receptors in the NAc. In the amygdala, the mRNA expression levels of kappa and delta opioid receptors were reduced after AEA administration, indicating the involvement of endocannabinoid- opioid cross-modulation in the taste preference of cows. Reduced *CNR1* gene expression in the left amygdala after repeated AEA administration was associated with changes in taste perception. This suggests that taste intensity perception is evaluated by the left amygdala in cows, which may reveal analogies to the perception of taste intensity in humans. However, further investigations are needed, e.g. the use of a selective CB1 antagonist, to validate the effect of AEA on taste preference. Knowledge about endocannabinoid-mediated changes in feed preference from late pregnancy to early lactation could contribute to the improvement of taste-dependent diet formulation and animal welfare.

## Material and methods

### Animals and housing

For this study, eight pairs of half-sib German Holstein cows at the end of the 1st or 2nd lactation from a local farm (Agrarprodukte Dedelow GmbH, Dedelow, Germany, the farm has consented to the use as experimental animals) were purchased. The half-sib pairs were comparable in lactation number, age (± 5 months), and expected calving date (± 6 days). Cows were pairwise transferred around Day 56 (± 17) before expected calving to the Experimental Facility for Cattle (FBN, Dummerstorf, Germany). Animals were housed in a free-stall barn with ad libitum access to water and feed, except during a maintenance period between 0500 and 0745 h. Cows were dried off 49 (± 20) days (d) a.p. except for one cow that was dried off 137 d before expected calving. After dry-off, cows were kept in a straw-bedding box until calving. All cows received three diets: a far-off diet after drying off until Day 25 (± 8) a.p., a close-up diet from Day 25 (± 8) a.p. until calving, and a lactation diet after calving (p.p.) (Fig. 1). All diets were offered as total mixed ration (TMR), and the nutrient composition was analysed (LUFA GmbH, Rostock, Germany) using near infrared spectroscopy according to VDLUFA (2004) (Supplementary Table 8). After calving, cows were transferred to the free-stall barn and milked twice daily at 0500 h and 1630 h.

### Grouping and treatment

**Figure 5:**
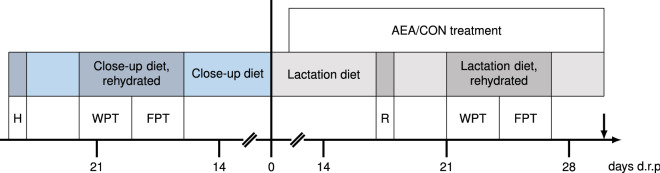
Scheme of the experimental procedure. On day 25 before expected calving, cows were habituated (H) to tie stalls and the experimental setup, this H procedure was repeated on Day 17 p.p as a reminder (R). The water preference test (WPT) was performed starting 22 days before expected calving and again 21 p.p. for three consecutive days. The feed preference test (FPT) was performed on day 19 before expected calving and on Day 24 p.p. for three consecutive days. During WPT and FPT, cows received a rehydrated close-up diet a.p. and a rehydrated lactation diet p.p. Intraperitoneal injections started on Day 1 p.p. and were continued until Day 30 p.p. from Mondays to Fridays. Cows received 3 µg/kg BW *N*-arachidonoylethanolamine (AEA, n = 8) or 0.9% NaCl, (CON; n = 8). Cows were slaughtered on Day 30 p.p. (arrow).

Each cow of a half-sib pair was randomly assigned to either the control (n = 8) or treatment group (n = 8; Fig. 5). After calving, the AEA group received intraperitoneal injections of 3 µg/kg bodyweight (BW) *N-*arachidonoylethanolamine (AEA; Tocris, Bristol, United Kingdom) diluted in 50 ml 0.9% NaCl. The AEA dose was calculated based on the BW determined at the day of calving, and the application dose remained constant over the course of the trial. A low dosage in the µg/kg range was based on Ackern et al^13^ and was chosen to avoid unwanted side effects which can occur in the mg/kg range in mice^86^. The CON group received 50 ml of 0.9% NaCl i.p. (CON). Cows that calved before 1400 h received the first injection on Day one p.p. and cows that calved after 1400 h on Day two p.p. Consecutive injections were performed daily from Mondays to Fridays, and additional injections were performed on Saturdays and Sundays during preference tests (see below). All injections were administered at 0700 h (± 34 min) until Day 30 (± 1) p.p. (25 days ± 2).

### Feed preparation for preference tests

The close-up and lactation diets were dried at 50–60 °C to evaporate volatile acids and other organic compounds potentially influencing taste. Based on the weight loss, the feed dry matter (DM) content was determined. The nutrient composition was analysed as described above (Table 1). The dried feed was rehydrated with tap water immediately before feeding based the weight loss from its former organic matter (OM). For the FPT, three different diets were prepared: taste-unaltered feed (control), which was dried feed with no additional compounds except tap water, and two feeds with different tastes (umami and sweet), generated by dissolving 5.1 g monosodium glutamate/kg OM (99%, AJINOMOTO FOODS EUROPE SAS, Paris, France) or 30 g sucrose/kg OM (Nordzucker AG, Braunschweig, Germany), respectively, in the water used for rehydration of dried feed.

**Table 1:** Nutrient composition and energy content of the dried close-up and lactation diet fed during the preference tests.

Item	Close-up diet		Lactation diet	
Nutrients, g/kg of DM	Mean	SD	Mean	SD
Crude ash	62.4	6.3	63.7	7.4
Crude protein	147.6	8.8	159.8	10.2
Crude fat	25.5	1.7	29.8	1.6
Crude fiber	189.3	17.3	157.8	7.9
Starch	234.4	37.6	266.0	23.0
Sugar	16.8	7.2	24.0	6.1
DM, %	34.9	4.0	39.0	3.1
ME, MJ/kg DM	10.5	0.4	11.3	0.2
NEL, MJ/kg DM	6.3	0.3	6.9	0.1

*DM* dry matter.

### Experimental setup

Prior to the preference test, cows received forced feeding. Cows were offered 5 kg of either umami- or sweet-tasting feed on two consecutive days between Day 36 and 23 a.p. During the test, cows were kept in tie stalls, which were equally equipped with a rubber flooring, horizontal fixation chain and a drinking bowl connected to the pressurized water system (referred to as the lateral drinking bowl). The water intake from the lateral bowl could not be measured. The preference test was set up in three parts and replicated a.p. and p.p. It was conducted with habituation on Day 25 (± 9) a.p. and the reminder on Day 17 (± 3) p.p. The habituation/reminder could not be performed for the first cow pair but was conducted on the remaining cows, with the WPT setting occurring from 0800 to 1200 h, followed by the FPT setting from 1200 to 1600 h. During the habituation/reminder, neither water nor feed was taste-altered. The water intake from one cow (AEA group) from the left drinking bowl during habituation could not be recorded due to technical issues. This cow was excluded from the statistical analysis of the water intake during habituation. Two days after habituation/reminder, the WPT and subsequently the FPT were performed between 0800 and 1600 h. The setting for the WPT and FPT consisted of a wooden rack (whd: 125 × 24 × 77 cm) placed in front of the cow. It was loaded with either drinking bowls or feeding bins containing the taste-altered water or feed, respectively, on the right and left side and a feed bin in between containing taste-unaltered feed (control feed). The right and left drinking bowl contained a low-pressure float valve and was connected via a hose to an elevated water barrel. Water intake was measured using scales (PCE-PB 150N, PCE Produktions- und Entwicklungsgesellschaft mbH, Meschede, Germany) placed underneath the barrels. Feed consumption from each bin was manually measured by hourly weighing.

### Water and feed preference test

The WPT started on Day 22 (± 9) a.p. and was replicated on Day 21 (± 3) p.p. Each test lasted eight hours on three consecutive days. During the WPT, cows chose between taste-unaltered water (lateral drinking bowl), umami-tasting water, and sweet-tasting water (left or right drinking bowl). Taste was added to the water by dissolving either 1.7 g/l monosodium glutamate (umami) or 0.18 g/l saccharin (sweet) (Saccharin, Buxtrade GmbH, Buxtehude, Germany). Saccharin, as a nonnutritive sweetener, was used in the WPT, as there is no risk of ruminal acidosis even with very high water intake. The FPT followed the WPT and was performed on Day 19 (± 9) a.p. and Day 24 (± 3) p.p. The setting was equivalent to the WPT except that the bowls were replaced by bins (left or right bin), and the feed was offered ad libitum. The respective position of taste-altered water or feed was assigned pseudorandomly on a day-to-day basis for each subject using the PROC PLAN statement in SAS to exclude lateral preference effects^45^.

### Video recording and behavioural analysis

The habituation/reminder, WPT, and FPT were recorded on video for behavioural analysis. The video cameras (HC-V777, Panasonic Corporation, Kadoma, Japan) were placed at an elevated position directly opposite to each tie stall. Video analysis was performed by five observers using the Observer XT 14 software (Noldus Information Technology BV, Wageningen, Netherlands). The behavioural analysis started when the cow entered the tie stall and ended at 1600 h. The total duration and total number of events in which the cow’s head entered the bowl or bin (defined as the moment when the cow’s muzzle crossed the upper horizontal plane of one of the bowls/bins) were analysed for habituation/reminder, WPT, and FPT. To evaluate interobserver-reliability, all observers analysed a three-hour video recording for WPT and FPT. The interobserver reliability was calculated to account for total duration and total number for both the WPT and FPT using the Fleiss Kappa test (WPT: duration: 0.989, frequency/sequence: 0.747, FPT: duration: 0.987, frequency/sequence: 0.814).

Due to technical difficulties, the video data were lacking for the 3rd FPT Day a.p. for one animal (cow 3) and the 1st FPT Day p.p. for another animal (cow 4). In addition, 234 min of video material for one animal (cow 1) on the 3rd FPT Day a.p. and 7 min of video material on the 1st WPT Day a.p. for two animals (cows 9 and 10) were lacking.

### Blood sampling and analyses

A blood sample was drawn from the jugular vein on day + 21 (± 1), 30 min after the i.p. injection in EDTA-containing tubes (Sarstedt AG & Co. KG, Nümbrecht, Germany) and was immediately placed on ice. Subsequently, blood samples were centrifuged at 1573×*g* for 20 min at 4 °C and the obtained plasma was stored at − 80 °C for further analysis. The concentration of plasma AEA was measured by using a triplequad mass spectrometer coupled HPLC method (LIPIDOMIX GmbH, Berlin, Germany). The analysis of the plasma leptin was performed with an enzyme immunoassay as previously described by Sauerwein et al. (2004)^87^.

### Real-time PCR (qPCR)

On Day 30 (± 1) p.p., cows were sacrificed by captive bolt stunning and subsequent exsanguination between 0800 and 0830 h at the institutional abattoir. Approximately 1 cm caudal of the tip, an approx. 2.5 cm × 4 cm piece of dorsal tongue epithelium was taken, snap frozen in liquid nitrogen and stored at − 80 °C for further analysis. The tissue was mortared in liquid nitrogen, and approximately 18 mg powdered tissue was subjected to RNA extraction, cDNA synthesis and qPCR as described recently with the following modification^88^. The PCR contained 2 µl cDNA (10 ng/µl), 1 µl H_2_O, 0.5 µl of each primer (4 µM), and 6 µl 2 × Puffer SensiFAST SYBR No-ROX mix (Bioline, London, UK). Data were quantified by qbase software (Biogazelle) using peptidylprolyl isomerase A (*PPIA*), emerin (*EMD)* and hypoxanthine phosphoribosyltransferase 1 (*HPRT1*) as reference genes (M-value: 0.215, CV-value: 0.088). The primer sequences are shown in Supplementary Table 9. The efficiency of amplification was calculated using LinReg-PCR software (v.2014.4, Academic Medical Centre, Amsterdam, Netherlands), yielding values between 1.74 and 1.90.

Additionally, the NAc and the corpus amygdaloideum were taken from the left and right hemispheres of the brain and stored in RNAlater (Qiagen, Hilden, Germany) at − 70 °C until analysis. The localization of these areas was determined using a stereotaxic atlas of the cow’s brain^89^. It was not possible to extract tissue from the left NAc of two animals and from the right NAc of one animal in the CON group. RNA extraction, cDNA synthesis and qPCR were performed as described by Kalbe and Puppe (2010) and Kalbe et al. (2018)^76,90^. Data are expressed as arbitrary units after normalization with the endogenous reference gene hydroxymethylbilane synthase (*HMBS*), which was not affected by treatment (*P* = 0.973 NAc, *P* = 0.664 amygdala). The primer sequences are shown in Supplementary Table 10.

### Statistical data analysis

The 3-day average of total duration, total number, total DMI and total water intake for each taste of the respective preference tests a.p. and p.p. was computed for the individual cows. For the habituation and reminder days, as well as for the preference tests, the percentages of total duration (TD), total number (TN), water intake and total DMI were calculated. All statistical analyses were performed using R Statistical Software (v4.2.0; R Core Team 2021, R Foundation for Statistical Computing, Vienna, Austria). Outliers were detected by using cooks distance (olsrr package, v0.5.3; Hebbali 2020) and visual inspection of boxplots. One outlier in the dataset of *GPR55* expression in tongue epithelia and one in the dataset of *OPRK1* expression in the left amygdala were detected. Outliers were excluded from the subsequent statistical analysis.

Data were analysed with a linear mixed model (LMM, lmer function, lme4 package, v1.1-29; Bates, Maechler, Walker 2015^91^). Based on the experimental design, sire were defined as random effect, despite the partly very low variance that can be assigned (0–60%). Random effects of the individual cows were not explicitly defined, as they are already contained in the residual variance. For habituation/reminder, “position of bin/bowl” (level: right, left, middle/lateral) was implemented as a fixed effect for the analyses of TD, TN, water intake and DMI, with the exception of water intake during habituation (see below). For the preference test, the “group” (level: AEA and CON), “taste” (level: sweet, umami, control) and their interaction were defined as fixed effects. For mRNA expression analysis of tissue obtained from the amygdala and nucleus accumbens, the fixed effects were “group” (level: AEA and CON), “hemisphere” (level: left and right) and the interaction (group × hemisphere). To analyse the mRNA expression in the tongue epithelium and the AEA and leptin plasma concentrations, “group” (level: AEA and CON) was the only tested fixed effect.

The residuals of all models were evaluated for normal distribution and homoscedasticity (check_normality and check_heteroscedasticity function, performance package, v0.9.0; Lüdecke et al.^92^). If the assumption of normality was violated, data were transformed with the Johnson transformation. The Wilcoxon signed rank test was used to analyse water intake during habituation, as no normal distribution of the residuals could be achieved using transformation. Nonconstant error variance was detected for the following data: *OPRK1* (amygdala), *OPRD1* (amygdala), DMI (habituation, reminder, FPT), TD (habituation feed setup, reminder feed setup, FPT + WPT a.p. + p.p.), TN (reminder feed setup, habituation water setup, FPT a.p. + p.p. and WPT a.p.) and AEA plasma concentration. However, heteroscedasticity has only a marginal impact on model estimates, allowing the models to be used despite the violation of the constant error variance^93^. Pairwise differences between levels of fixed effects were tested by using the Tukey Kramer test. For the fixed effect of interest, estimated marginal means and their standard errors (SEs) were estimated. Effects and differences were considered significant at *P* < 0.05. For interpretation purposes, the means of the observed data and their *standard deviations* (SDs) are presented in the figures.

### Ethical note

The experimental protocol was approved by the Federal Office of Agriculture, Food Security and Fishery Mecklenburg-Western Pomerania, Rostock, Germany (LALLF MV/TSD/7221.3-1-015/19) and conducted in accordance with ARRIVE guidelines (https://arriveguidelines.org), the European Directive 2010/63/EU, the German Animal Welfare Act and the German Regulation on the Protection of Animals in Connection with Slaughter or Killing and on the Implementation of Council Regulation (EC) No 1099/2009.

## Supplementary Information

Supplementary Information.

## Data Availability

All data generated and analysed are available on request from the corresponding author.
