# Supplementary material for: Endocannabinoid administration affects taste preference and the expression of cannabinoid and opioid receptors in the amygdala of early lactating cows
Source: Sci Rep. 2023 Mar 27;13:4967. doi: 10.1038/s41598-023-31724-3 (PMC10042870; doi:10.1038/s41598-023-31724-3)
Supplement: Supplementary file 1 — Supplementary Information. [file 41598_2023_31724_MOESM1_ESM.docx]

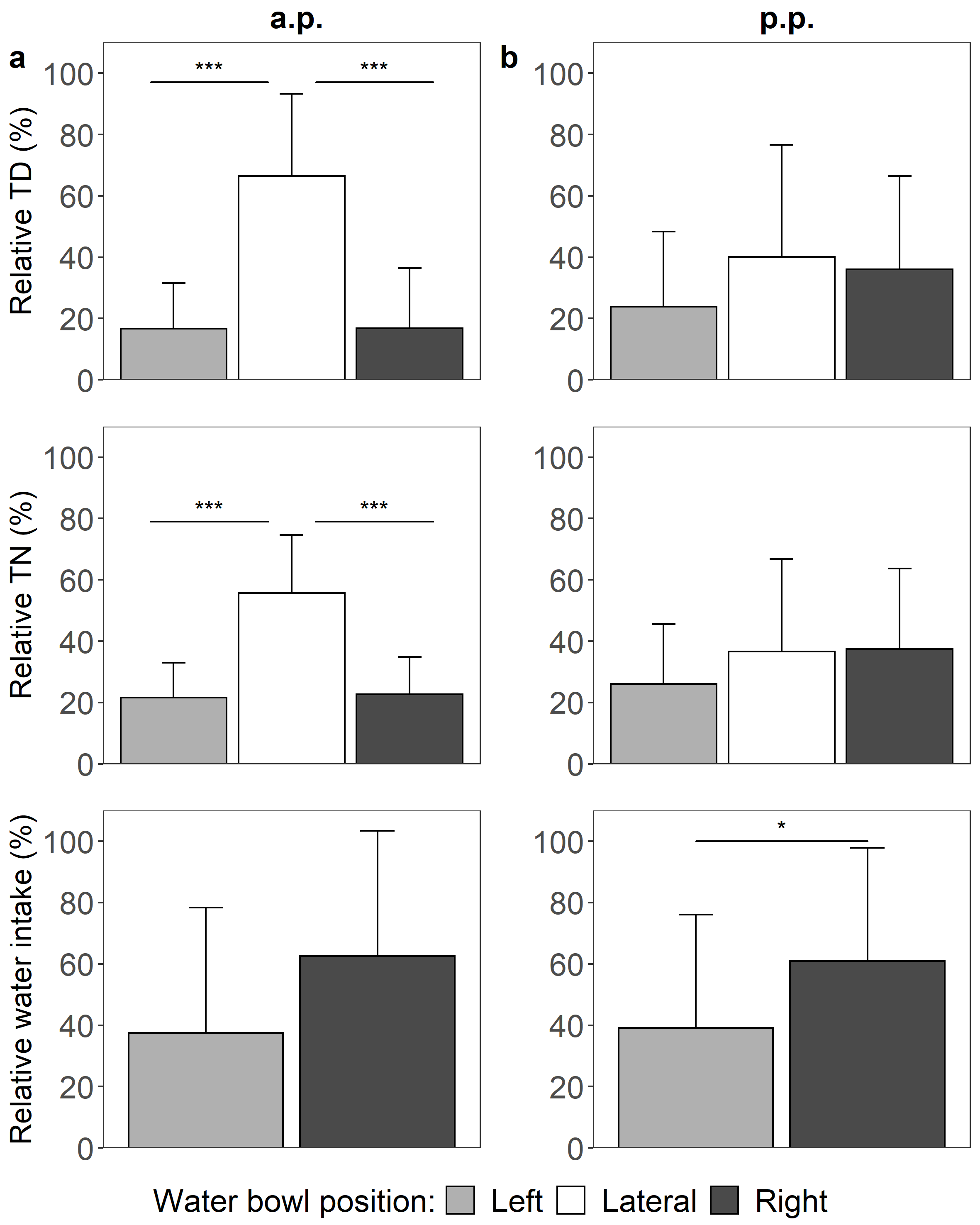
 **Supplementary Figure 1.** Percentage of total duration (TD) and total number (TN) of events the head entered the drinking bowl, and percentage of water intake from different positions (**a**) during the 4-h habituation period in week 4 antepartum (a.p.), and (**b**) during the 4-h reminder period in week 3 postpartum (p.p.). Two drinking bowls were located on the left and right in front of the cow (n=16), and one trough was mounted laterally. Water intake from the lateral bowl was not measured. The mean of the observed data ± SD is shown for interpretation. * P < 0.05, *** *P* < 0.001.


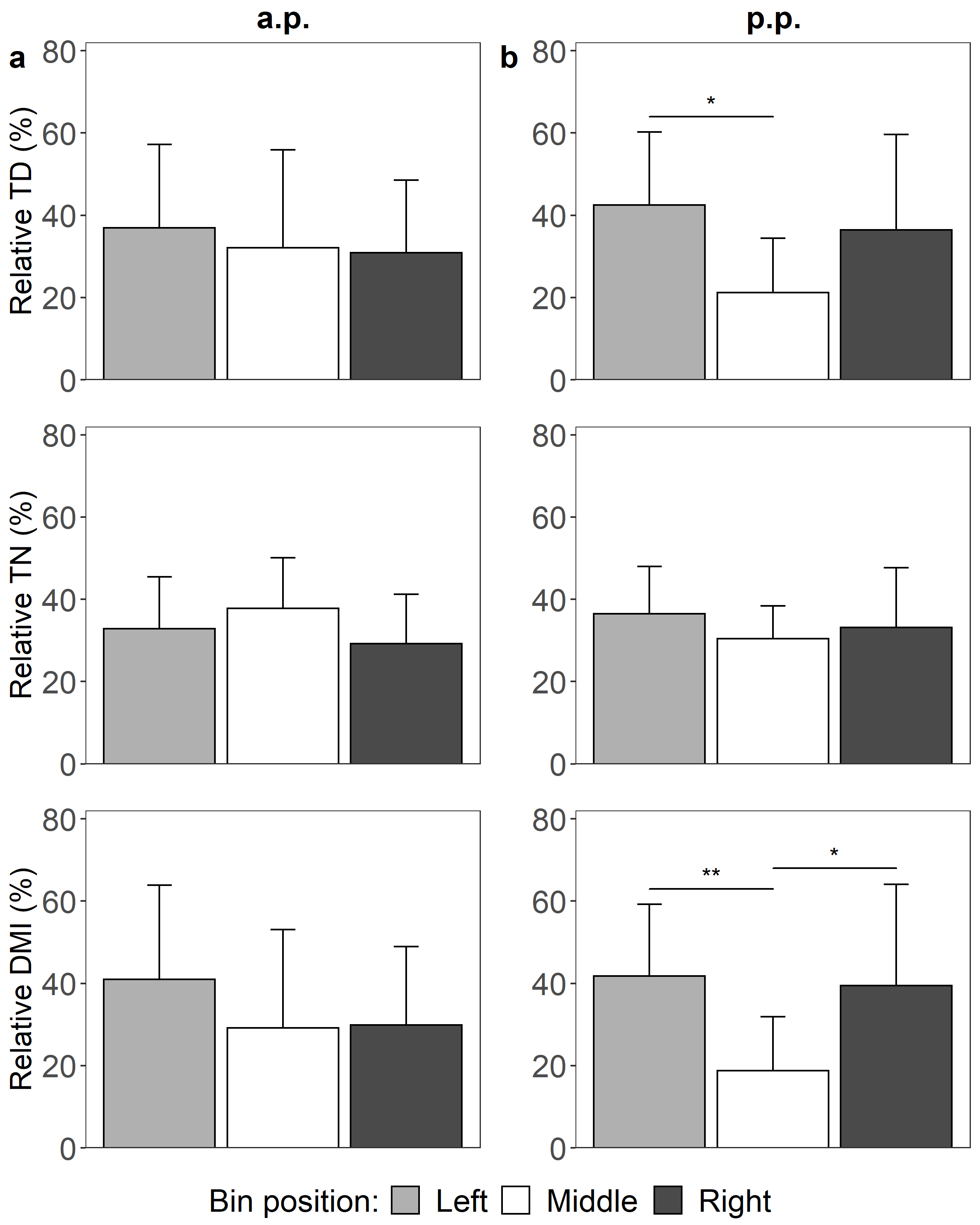


**Supplementary Figure 2.** Percentage of total duration (TD) and total number (TN) of events the head entered the feeding bin, as well as percentage of dry matter intake (DMI) from feeding bins placed on the right, left and middle position in front of the cow (n=16) (**a**) during the 4-h habituation period in week 4 antepartum (a.p.), and (**b**) on reminder day in week 3 postpartum (p.p.). The mean of the observed data ± SD is shown for interpretation. * *P* < 0.05, ** *P* < 0.01

**Supplementary Figure 3**


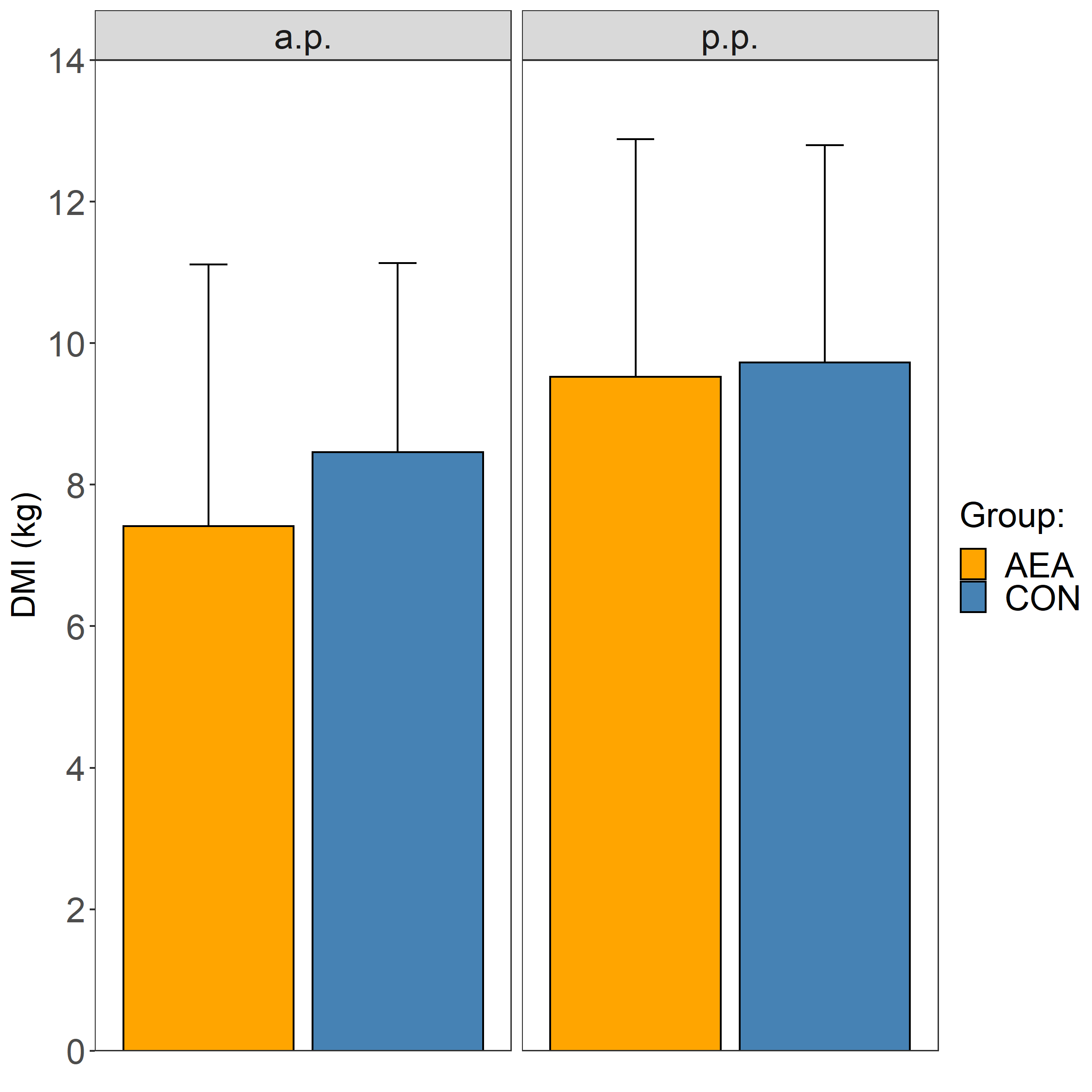


**Supplementary Figure 3.** Three-day average of dry matter intake (DMI) during the feed preference test during the antepartum (a.p.) and postpartum (p.p.) period. During the p.p. period, cows were treated intraperitoneally with NaCl (CON, n = 8) or *N*-arachidonoylethanolamide (AEA, n = 8).The mean of the observed data ± SD is shown for interpretation.


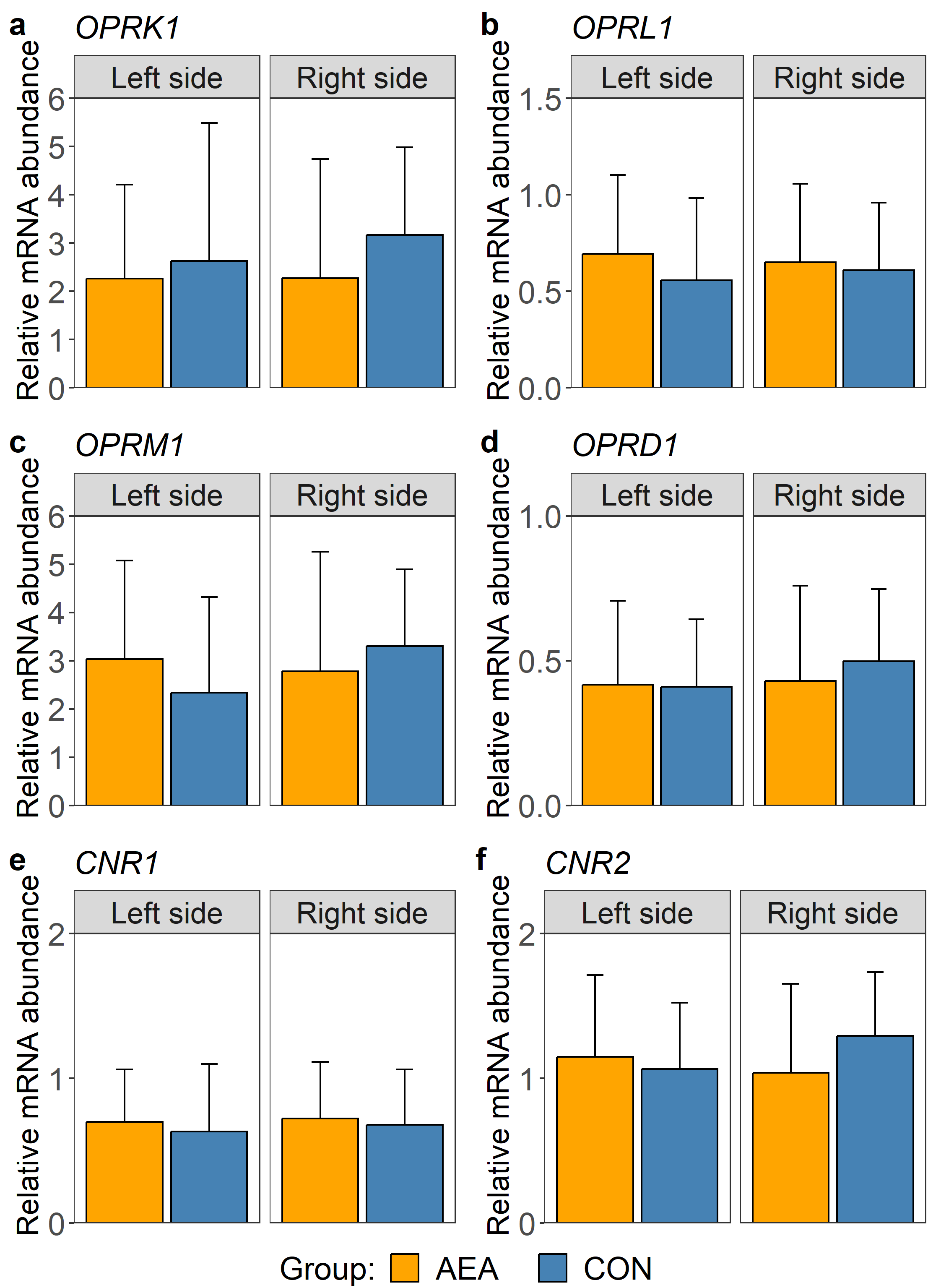


**Supplementary Figure 4.** Relative mRNA abundance of (**a**) opioid receptor kappa 1 (*OPRK1*), (**b**) opioid related nociceptin receptor 1 (*OPRL1*), (**c**) opioid receptor mu 1 (*OPRM1*), (**d**) opioid receptor delta 1 (*OPRD1*), (**e**) cannabinoid receptor 1 (*CNR1*) and **(f)** cannabinoid receptor 2 (*CNR2*) in left and right nucleus accumbens of cows treated intraperitoneally with NaCl (CON, left hemisphere n = 6, right hemisphere n = 7) or *N*-arachidonoylethanolamide (AEA, left hemisphere n = 8, right hemisphere n = 8). The mean of the observed data ± SD is shown for interpretation.

**
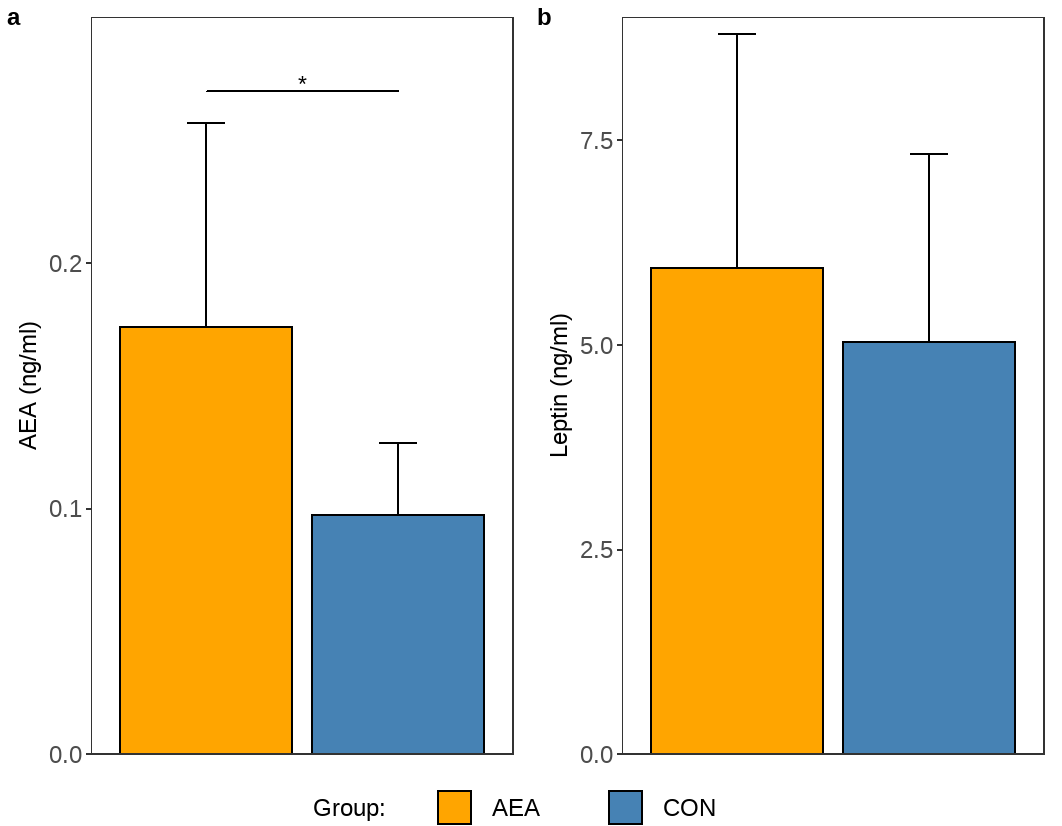
**

**Supplementary Figure 5.** Plasma concentration of *N*-arachidonoylethanolamide (AEA, **a**) and leptin (**b**) of cows treated intraperitoneally with *N*-arachidonoylethanolamide (AEA, n = 8) or NaCl (CON, n = 8). The mean of the observed data ± SD is shown for interpretation. * *P* < 0.05

**Supplementary Table 1.** ANOVA results of the habituation/reminder.

| **Habituation/Reminder** | **Value** | **Effect** | **Sum Sq** | **Num DF** | **Den DF** | **F value** | **P value** |
| --- | --- | --- | --- | --- | --- | --- | --- |
| Habituation FPT | DMI | Position | 1407.9 | 2 | 45 | 1.442 | 0.247 |
|  | TD | Position | 330.8 | 2 | 45 | 0.385 | 0.683 |
|  | TN | Position | 594.3 | 2 | 45 | 1.983 | 0.150 |
| Reminder FPT | DMI | Position | 4482.4 | 2 | 39 | 6.222 | 0.005 |
|  | TD | Position | 3368.7 | 2 | 39 | 4.899 | 0.013 |
|  | TN | Position | 259.5 | 2 | 39 | 0.952 | 0.395 |
| Habituation WPT | TD | Position | 26344.0 | 2 | 45 | 29.891 | <0.001 |
|  | TN | Position | 11964.0 | 2 | 45 | 27.982 | <0.001 |
| Reminder WPT | Water intake | Position | 5.6 | 1 | 26 | 4.776 | 0.038 |
|  | TD | Position | 1.9 | 2 | 39 | 1.093 | 0.345 |
|  | TN | Position | 1.3 | 2 | 39 | 0.637 | 0.534 |

Sum of squares (Sum Sq), numerator degrees of freedom (Num DF), denominator degrees of freedom (Den DF)

**Supplementary Table 2.** ANOVA results of the WPT

| **Preference test** | **Value** | **Effect** | **Sum Sq** | **Num DF** | **Den DF** | **F value** | **P value** |
| --- | --- | --- | --- | --- | --- | --- | --- |
| WPT a.p. | Water intake | Group | 0.0 | 1 | 28 | 0.000 | 1.000 |
|  | Water intake | Taste | 11240.8 | 1 | 28 | 29.655 | <0.001 |
|  | Water intake | Group x Taste | 949.8 | 1 | 28 | 2.506 | 0.125 |
|  | TD | Group | 0.0 | 1 | 42 | 0.000 | 1.000 |
|  | TD | Taste | 8505.7 | 2 | 42 | 5.100 | 0.010 |
|  | TD | Group x Taste | 112.5 | 2 | 42 | 0.067 | 0.935 |
|  | TN | Group | 0.0 | 1 | 42 | 0.000 | 1.000 |
|  | TN | Taste | 4199.2 | 2 | 42 | 3.762 | 0.031 |
|  | TN | Group x Taste | 107.7 | 2 | 42 | 0.097 | 0.908 |
| WPT p.p. | Water intake | Group | 0.0 | 1 | 28 | 0.000 | 1.000 |
|  | Water intake | Taste | 13.3 | 1 | 28 | 18.747 | <0.001 |
|  | Water intake | Group x Taste | 0.3 | 1 | 28 | 0.421 | 0.522 |
|  | TD | Group | 0.0 | 1 | 42 | 0.000 | 1.000 |
|  | TD | Taste | 10991.8 | 2 | 42 | 5.671 | 0.007 |
|  | TD | Group x Taste | 1740.4 | 2 | 42 | 0.898 | 0.415 |
|  | TN | Group | 0.3 | 1 | 42 | 0.303 | 0.585 |
|  | TN | Taste | 1.5 | 2 | 42 | 0.795 | 0.458 |
|  | TN | Group x Taste | 1.0 | 2 | 42 | 0.516 | 0.601 |

Sum of squares (Sum Sq), numerator degrees of freedom (Num DF), denominator degrees of freedom (Den DF)

**Supplementary Table 3.** ANOVA results of the FPT

| **Preference test** | **Value** | **Effect** | **Sum Sq** | **Num DF** | **Den DF** | **F value** | **P value** |
| --- | --- | --- | --- | --- | --- | --- | --- |
| FPT a.p. | DMI | Group | 0.0 | 1 | 42 | 0.000 | 1.000 |
|  | DMI | Taste | 13490.0 | 2 | 42 | 35.638 | <0.001 |
|  | DMI | Group x Taste | 127.0 | 2 | 42 | 0.335 | 0.717 |
|  | TD | Group | 0.0 | 1 | 42 | 0.000 | 1.000 |
|  | TD | Taste | 10684.6 | 2 | 42 | 36.622 | <0.001 |
|  | TD | Group x Taste | 94.5 | 2 | 42 | 0.324 | 0.725 |
|  | TN | Group | 0.0 | 1 | 42 | 0.000 | 1.000 |
|  | TN | Taste | 3073.9 | 2 | 42 | 23.401 | <0.001 |
|  | TN | Group x Taste | 103.6 | 2 | 42 | 0.789 | 0.461 |
| FPT p.p. | DMI | Group | 0.0 | 1 | 42 | 0.000 | 1.000 |
|  | DMI | Taste | 11672.0 | 2 | 42 | 12.703 | <0.001 |
|  | DMI | Group x Taste | 1124.0 | 2 | 42 | 1.223 | 0.305 |
|  | TD | Group | 0.0 | 1 | 42 | 0.000 | 1.000 |
|  | TD | Taste | 8877.8 | 2 | 42 | 10.224 | <0.001 |
|  | TD | Group x Taste | 787.2 | 2 | 42 | 0.907 | 0.412 |
|  | TN | Group | 0.0 | 1 | 42 | 0.000 | 1.000 |
|  | TN | Taste | 2783.4 | 2 | 42 | 5.912 | 0.006 |
|  | TN | Group x Taste | 482.7 | 2 | 42 | 1.025 | 0.368 |

Sum of squares (Sum Sq), numerator degrees of freedom (Num DF), denominator degrees of freedom (Den DF)

**Supplementary Table 4.** ANOVA results of tongue epithelia mRNA expression

| **Gene** | **Effect** | **Sum Sq** | **Num DF** | **Den DF** | **F value** | **P value** |
| --- | --- | --- | --- | --- | --- | --- |
| *GNAT3* | Group | 1.3 | 1 | 7 | 3.558 | 0.101 |
| *TASR2* | Group | 0.4 | 1 | 7 | 2.419 | 0.164 |
| *GPR55* | Group | 1.8 | 1 | 5 | 3.500 | 0.120 |
| *CNR1* | Group | 0.3 | 1 | 7 | 1.202 | 0.309 |

Sum of squares (Sum Sq), numerator degrees of freedom (Num DF), denominator degrees of freedom (Den DF)

**Supplementary table 5.** ANOVA results of nucleus accumbens mRNA expression.

| **Tissue** | **Gene** | **Effect** | **Sum Sq** | **Num DF** | **Den DF** | **F value** | **P value** |
| --- | --- | --- | --- | --- | --- | --- | --- |
| NAc | *OPRK1* | Group | 2.2233 | 1 | 18.10 | 0.674 | 0.422 |
|  |  | Hemisphere | 0.1689 | 1 | 18.34 | 0.051 | 0.824 |
|  |  | Group x hemisphere | 0.1429 | 1 | 18.34 | 0.043 | 0.838 |
|  | *OPRD1* | Group | 0.0028 | 1 | 18.30 | 0.041 | 0.842 |
|  |  | Hemisphere | 0.0183 | 1 | 18.69 | 0.265 | 0.613 |
|  |  | Group x hemisphere | 0.0104 | 1 | 18.69 | 0.151 | 0.702 |
|  | *OPRM1* | Group | 0.1174 | 1 | 18.00 | 0.059 | 0.811 |
|  |  | Hemisphere | 0.1684 | 1 | 18.16 | 0.084 | 0.775 |
|  |  | Group x hemisphere | 1.1461 | 1 | 18.16 | 0.572 | 0.459 |
|  | *OPRL1* | Group | 0.1147 | 1 | 17.95 | 1.368 | 0.257 |
|  |  | Hemisphere | 0.0017 | 1 | 18.14 | 0.020 | 0.890 |
|  |  | Group x hemisphere | 0.0060 | 1 | 18.14 | 0.072 | 0.792 |
|  | *CNR1* | Group | 0.0672 | 1 | 18.15 | 0.839 | 0.372 |
|  |  | Hemisphere | 0.0047 | 1 | 18.33 | 0.059 | 0.812 |
|  |  | Group x hemisphere | 0.0000 | 1 | 18.33 | 0.000 | 0.983 |
|  | *CNR2* | Group | 0.0520 | 1 | 18.53 | 0.230 | 0.637 |
|  |  | Hemisphere | 0.0263 | 1 | 18.86 | 0.117 | 0.737 |
|  |  | Group x hemisphere | 0.2088 | 1 | 18.86 | 0.924 | 0.349 |

Sum of squares (Sum Sq), numerator degrees of freedom (Num DF), denominator degrees of freedom (Den DF)

**Supplementary table 6**. ANOVA results of amygdala mRNA expression

| **Tissue** | **Gene** | **Effect** | **Sum Sq** | **Num DF** | **Den DF** | **F value** | **P value** |
| --- | --- | --- | --- | --- | --- | --- | --- |
| Amygdala | *OPRK1* | Group | 0.0641 | 1 | 27 | 6.879 | 0.014 |
|  |  | Hemisphere | 0.0003 | 1 | 27 | 0.034 | 0.855 |
|  |  | Group x hemisphere | 0.0044 | 1 | 27 | 0.475 | 0.497 |
|  | *OPRD1* | Group | 0.3140 | 1 | 28 | 5.702 | 0.024 |
|  |  | Hemisphere | 0.1554 | 1 | 28 | 2.822 | 0.104 |
|  |  | Group x hemisphere | 0.0587 | 1 | 28 | 1.065 | 0.311 |
|  | *OPRM1* | Group | 0.0001 | 1 | 28 | 0.001 | 0.980 |
|  |  | Hemisphere | 0.1035 | 1 | 28 | 0.603 | 0.444 |
|  |  | Group x hemisphere | 0.0000 | 1 | 28 | 0.000 | 1.000 |
|  | *OPRL1* | Group | 3.1678 | 1 | 21 | 6.992 | 0.015 |
|  |  | Hemisphere | 0.0619 | 1 | 21 | 0.137 | 0.715 |
|  |  | Group x hemisphere | 0.0102 | 1 | 21 | 0.022 | 0.882 |
|  | *CNR1* | Group | 1.0989 | 1 | 21 | 10.038 | 0.005 |
|  |  | Hemisphere | 0.2195 | 1 | 21 | 2.005 | 0.172 |
|  |  | Group x hemisphere | 0.1093 | 1 | 21 | 0.998 | 0.329 |
|  | *CNR2* | Group | 0.0465 | 1 | 28 | 0.852 | 0.364 |
|  |  | Hemisphere | 0.2145 | 1 | 28 | 3.928 | 0.057 |
|  |  | Group x hemisphere | 0.0231 | 1 | 28 | 0.423 | 0.521 |

Sum of squares (Sum Sq), numerator degrees of freedom (Num DF), denominator degrees of freedom (Den DF)

**Supplementary table 7**. ANOVA results of AEA and leptin plasma concentration

| **Tissue** | **Effect** | **Sum Sq** | **Num DF** | **Den DF** | **F value** | **P value** |
| --- | --- | --- | --- | --- | --- | --- |
| AEA | Group | 0.0232 | 1 | 7 | 9.694 | 0.017 |
| Leptin | Group | 0.7923 | 1 | 14 | 0.815 | 0.382 |

Sum of squares (Sum Sq), numerator degrees of freedom (Num DF), denominator degrees of freedom (Den DF)

**Supplementary Table 8:** Feed constituents, nutrient composition and energy content of far-of, close-up and lactation diet (mean ± SD).

| **Item** | **far-off** | | | **close-up** | | **lactation** | | |
| --- | --- | --- | --- | --- | --- | --- | --- | --- |
| **Component, g/kg of DM** | | **mean** | **SD** | **mean** | **SD** | | **mean** | **SD** |
| Grass/alfalfa-silage | | 512.8 | 215.5 | 242.3 | 100.4 | | 214.4 | 69.2 |
| Corn silage | | 107.6 | 73.2 | 342.9 | 39.4 | | 379.7 | 37.2 |
| Rye silage | | 134.8 | 175.5 | 40.0 | 49.0 | | 12.2 | 20.3 |
| Hay | | 67.0 | 20.3 | 61.8 | 20.3 | |  |  |
| Straw | | 170.1 | 64.8 | 59.8 | 22.5 | | 31.9 | 10.8 |
| Rapeseed extraction meal | |  |  | 41.4 | 18.6 | | 46.0 | 6.0 |
| Soybean extraction meal | |  |  | 26.4 | 33.8 | | 22.9 | 13.7 |
| Wheatmeal | |  |  | 57.5 | 5.6 | | 16.6 | 1.7 |
| Corn meal | |  |  |  |  | | 50.8 | 17.2 |
| Milk performance feed^1^ | |  |  | 113.8 | 37.6 | | 215.0 | 15.5 |
| Mineral feed^2^ | | 7.7 | 0.6 |  |  | | 6.4 | 0.7 |
| Mineral feed^3^ | |  |  | 14.1 | 1.4 | |  |  |
| Limestone | |  |  |  |  | | 3.3 | 0.9 |
| Soybean oil | |  |  |  |  | | 0.9 | 0.2 |
|  | |  |  |  |  | |  |  |
| **Nutrients, g/kg of DM** | |  |  |  |  | |  |  |
| Crude ash | | 82.1 | 5.1 | 59.1 | 4.5 | | 58.5 | 54.8 |
| Crude protein | | 131.8 | 4.4 | 147.4 | 5.5 | | 164.4 | 12.0 |
| Crude fat | | 19.5 | 3.3 | 25.7 | 1.2 | | 30.7 | 1.3 |
| Crude fiber | | 265.7 | 13.4 | 191.7 | 10.9 | | 153.9 | 8.2 |
| Starch | | 91.7 | 16.5 | 226.3 | 11.1 | | 278.6 | 24.8 |
| Sugar | | 4.0 | 6.9 | 13.4 | 8.0 | | 21.0 | 7.8 |
|  | |  |  |  |  | |  |  |
|  | |  |  |  |  | |  |  |
| DM content, % | | 33.8 | 3.3 | 39.0 | 2.5 | | 41.2 | 2.8 |
| ME, MJ/kg DM | | 8.5 | 0.5 | 10.5 | 0.2 | | 11.4 | 0.2 |
| NE_L_, MJ/kg DM | | 4.9 | 0.4 | 6.3 | 0.2 | | 7.0 | 0.1 |

Dry matter (DM)

^1^ MF2000 (Ceravis AG, Rendsburg, Germany): Soybean extraction meal from hulled seed; steam-heated, wheat, corn, canola extraction meal, beet molasses pulp, malt germ, dried stillage (grain), beet molasses, sodium bicarbonate, beet vinasse, calcium carbonate, sodium chloride, calcium-sodium phosphate, 24% crude protein, 2.6% crude fat, 5.1% crude fiber, 8% crude ash, 0.73% calcium, 0.5% phosphorous, 0.65% sodium, 7.1MJ NEL/kg; Additives per kg organic matter: 10.000 I.U vitamin A, 1.125 IU vitamin D, 40 mg vitamin E, 0.6 mg I, 0.4 mg Co, 50 mg Mn, 75 mg Zn, 0.4 mg Se.

^2^Panto Mineral R 8609 (HL Hamburger Leistungsfutter GmbH, Hamburg, Germany): 20% calcium, 6% phosphorous, 8% sodium, 6% magnesium, 0.03% inorganic nitrogen, 13.7% phosphorous pentoxide; Additives per kg original substance: 900.000 IU vitamin A, 200.000 IU vitamin D3, 4.500 mg vitamin E, 1.5 g Cu, 8 g Zn, 5 g Mn, 60 mg I, 21 mg Co, 50 mg Se.

^3^KULMIN MFV Plus (Bergophor Futtermittelfabrik Dr. Berger GmbH & Co. KG, Kulmbach, Germany): 0.7% calcium, 5.5% phosphorous, 10% magnesium, 5% sodium, 3.5% HCl-insoluble ash; Additives per kg organic matter: 850000 IU vitamin A, 200000 IU vitamin D3, 8000mg vitamin E, 200 mg vitamin B1, 80 mg vitamin B2, 100 mg vitamin B6, 25.000 mg vitamin B12, 200 mg vitamin B5, 1000 mg niacin amide. 10.0000 mg biotin, 10.000 mg choline chloride, 1.000mg Cu, 5.000 mg Zn, 3.000mg Mn. 20 mg Co, 75 mg I, 45 mg Se. 14.0 *10^19 CFU Saccharomyces cerevisiau, 75 mg propyl gallate, tocopherol excract of plant oils, citric acid, 40.000 mg flavoring blend with 8.5% polyphenol content.

**Supplementary Table 9.** Primer sequences, PCR product sizes and amplification efficiency for genes analysed in tongue epithelium.

| **Gene** | **Function** | **Primer sequence (5´to 3´)** | **Accession ID** | **Size (bp)** | **T_A_ (C°)** |
| --- | --- | --- | --- | --- | --- |
| *EMD* | Forward | GCCCTCAGCTTCACTCTCAGA | NM_203361 | 100 | 60 |
|  | Reverse | GAGGCGTTCCCGATCCTT |  |  |  |
| *PPIA* | Forward | GGATTTATGTGCCAGGGTGGTGA | NM_178320 | 120 | 60 |
|  | Reverse | CAAGATGCCAGGACCTGTATG |  |  |  |
| *HPRT1* | Forward | CTACTGAAACACTGGCGGGAC | NM_001034035 | 108 | 60 |
|  | Reverse | TACTGCTACTGTGTGCTTAGG |  |  |  |
| *GNAT3* | Forward | GCTTTGAAAGGGCATCTGAA | NM_001109982 | 235 | 60 |
|  | Reverse | CCTTCAAAGCAGTGAATCCAT |  |  |  |
| *TAS1R2* | Forward | GTACAGCGCCATCAACGAC | NM_001206529 | 109 | 60 |
|  | Reverse | CAGCAGCATCTGCACTATGG |  |  |  |
| *CNR1* | Forward | AAGCCCGCATGGACATTCGGCTGG | NM_001242341 | 79 | 60 |
|  | Reverse | AGCAGAGGGCCCCAGCAGAT |  |  |  |
| *GPR55* | Forward | TGCTGCCTGGATGTTTTCTG | XM_024982054 | 207 | 60 |
|  | Reverse | TCAGCCCACCACATCAGG |  |  |  |

T_A_, annealing temperature

Emerin (*EMD),* peptidylprolyl isomerase a (*PPIA*), hypoxanthine phosphoribosyltransferase 1(*HPRT1*), g protein subunit alpha transducin 3 (*GNAT3*), taste 1 receptor member 2 (*TAS1R2*), cannabinoid receptor 1 (*CNR1*), G protein-coupled receptor 55 (*GPR55*)

**Supplementary Table 10.** Primer sequences, PCR temperature conditions and product sizes for the analysis of genes analysed in the amygdala and nucleus accumbens.

| **Gene** | **Function** | **Primer sequence (5´to 3´)** | **Accession ID/Reference** | **Size (bp)** | **T_A_ (C°)** |
| --- | --- | --- | --- | --- | --- |
| *HMBS* | Forward | CTGTTTACCAAGGAGCTGGAAC | Erkens et al. 2006^94^ | 100 | 60 |
|  | Reverse | TGAAGCCAGGAGGAAGCA |  |  |  |
| *OPRL1* | Forward | CCAAGGGAGTCAGGGTCTCT | NM_001318971 | 128 | 60 |
|  | Reverse | GCAGGTGCTCTTGGTAGAGG |  |  |  |
| *OPRM1* | Forward | TGCCTCAACCCCGTCCTTTA | NM_174408 | 201 | 60 |
|  | Reverse | CGGAGTGGTTTCTGCTTCCAG |  |  |  |
| *OPRK1* | Forward | TCTACTCCGTGGTGTTCGTC | NM_001046480 | 150 | 60 |
|  | Reverse | GGAAGGGCATAGTTGTGGTCA |  |  |  |
| *OPRD1* | Forward | CAACGTGCTCGTCATGTTCG | NM_001191148 | 188 | 60 |
|  | Reverse | AGTCAATGGAGAGCACGACC |  |  |  |
| *CNR1* | Forward | AAGCCCGCATGGACATTCGGCTGG | NM_001242341 | 79 | 60 |
|  | Reverse | AGCAGAGGGCCCCAGCAGAT |  |  |  |
| *CNR2* | Forward | TCTTCGCCGGCATCATCTAC | NM_001192303 | 110 | 60 |
|  | Reverse | CATCCGGGCTATTCCAGACA |  |  |  |

T_A_, annealing temperature, hydroxymethylbilane synthase (*HMBS*), opioid related nociceptin receptor 1(*OPRL1*), opioid receptor mu 1 (*OPRM1*), opioid receptor kappa 1 (*OPRK1*), opioid receptor delta 1 (*OPRD1*), cannabinoid receptor 1 (*CNR1*), cannabinoid receptor 2 (*CNR2*). ^94^Erkens, T. et al. Development of a new set of reference genes for normalization of real-time RT-PCR data of porcine backfat and longissimus dorsi muscle, and evaluation with PPARGC1A. BMC Biotechnol 6, 41, doi:10.1186/1472-6750-6-41 (2006).
